# Microbial players in autoimmunity: multicentric analysis of the association between *Mycoplasma hominis* serostatus and rheumatoid arthritis

**DOI:** 10.1128/spectrum.01477-24

**Published:** 2025-02-04

**Authors:** G. L. Erre, Ngoc Dan Thanh Phan, N. Diaz, A. Congiargiu, N. Mundula, A. A. Mangoni, Thi Minh Phuong Phan, V. Margarita, P. L. Fiori, P. Rappelli, C. Cacciotto, A. Alberti, D. Dessì

**Affiliations:** 1 Dipartimento di Medicina, Chirurgia e Farmacia, Università degli Studi di Sassari, Sassari, Italy; 2 UO Reumatologia, Azienda Ospedaliero-Universitaria di Sassari, Sassari, Italy; 3 Hue University of Medicine and Pharmacy, Hue, Vietnam; 4 Dipartimento di Scienze Biomediche, Università degli Studi di Sassari, Sassari, Italy; 5 Department of Clinical Pharmacology, College of Medicine and Public Health, Flinders University and Flinders Medical Centre, Adelaide, Australia; 6 Dipartimento di Medicina Veterinaria, Università degli Studi di Sassari, Sassari, Italy; Southern Medical University, Guangzhou, China

**Keywords:** autoimmunity, *Mycoplasma hominis*, rheumatoid arthritis, immunoserology

## Abstract

Resident mucosal pathogens may induce immune tolerance breach, specific autoimmune response, and the development of rheumatoid arthritis (RA) in susceptible individuals. Despite a number of studies linking infections by *Mollicutes* bacteria to autoimmune disorders’ onset and progression, the role of *Mycoplasma hominis*, a common urogenital mucosa colonizing bacterium, in inducing a specific humoral response in RA has been seldom addressed. This study reports *M. hominis* seroprevalence in RA patients compared to healthy controls (HC) by testing two separate cohorts sampled in two distinct geographical settings (Italy and Vietnam). The amount of circulating anti-lipid-associated membrane proteins (LAMPs) antibodies was significantly higher in RA patients than HC in both cohorts. Also, a significantly higher seroprevalence of anti-*M*. *hominis* antibodies in RA patients compared to HC in both cohorts was observed. Notably, neither ELISA OD values nor positivity of anti-LAMPs were significantly associated with RA-specific variables. Further studies are essential to elucidate the underlying mechanisms by which *Mycoplasma* species may contribute to the pathogenesis of RA, thereby advancing our understanding of the potential causal links between *Mollicutes* and autoimmune disorders.

**IMPORTANCE:**

Mycoplasmas may cause persistent asymptomatic mucosal infections and elicit chronic host immune responses. In this study, we evaluated the prevalence of serological response to the sexually transmitted bacterium *Mycoplasma hominis* in patients with rheumatoid arthritis. We show that sera of patients with rheumatoid arthritis are enriched with antibodies specifically recognizing microbial surface antigens compared with the general population. This suggests that *M. hominis* genital infection, with its peculiar host immunity subversion mechanisms, might play a role in predisposing to the development and progression of chronic arthritis in susceptible individuals. Thus, the range of microbes with a role as triggers of autoimmune disease (*P. gingivalis*, A. *actinomycetemcomitans*, *Streptococcus* spp., and *F. nucleatum*, among others) might have a new member in *M. hominis*. The potential role of the interactions taking place at the host–pathogen interface during persistent *M. hominis* infections in inducing autoimmunity should be further explored and characterized.

## OBSERVATION

Mycoplasmas are a composite group of microorganisms characterized by minimal genomes and hence by limited biosynthetic capabilities, which evolutionarily lead to an obligate reliance on a host. As a consequence of this strict dependence, many *Mycoplasma* species evolved as pathobionts establishing long-lasting chronic infections often resulting in a number of diseases in a wide range of plant and animal species, including humans (1). This peculiar condition has led mycoplasmas to develop a range of strategies allowing survival, including the subversion of immune responses by inducing nonspecific suppressive or stimulatory effects on immune cells (2). The ability of mycoplasmas to elicit anti- and pro-inflammatory cytokines may lead to perturbation of host immune homeostasis, and hence to autoimmune disease in susceptible subjects. Indeed, *M. pneumoniae* extrapulmonary manifestations are believed to be linked to several autoimmune conditions (3, 4), while an increased prevalence of *M. orale* infection has been related to Sjögren syndrome (5).

Rheumatoid arthritis (RA) is a common systemic autoimmune disease affecting ~2% of the global population, characterized by chronic inflammation (6, 7). Although the exact mechanisms underpinning the pathogenesis of RA are unknown, current knowledge suggests a combination of specific genetic susceptibility and several environmental factors, including microbial infections, promoting loss of tolerance, and specific autoimmune response (6, 8, 9).

While most of the investigations exploring the role of microorganisms as environmental triggers of RA are largely associative, a number of recent studies focus on possible mechanisms such as protein citrullination, molecular mimicry of RA-relevant antigens, interferon-alpha production, and autoreactive lymphocyte clone selection (10). In addition, neutrophils appear to contribute to inflammation and citrullination at mucosal sites through the release of neutrophil extracellular traps (NETs), complexes of DNA, histones, and antimicrobial peptides (11). Furthermore, the citrullinating enzyme protein arginine deiminase (PAD) can be released in the context of NETs and subsequently activated by extracellular Ca^2+^, promoting citrullination of extracellular proteins and contributing to RA-specific autoimmune response (12, 13). Of note, the presence of anti-citrullinated protein autoantibodies (ACPA), a hallmark feature of RA, in the vaginal fluid of RA females in premenopausal age supports the hypothesis that some urogenital mucosal pathobiont may play a role in stimulating a specific humoral response, NETs generation, and predisposition to RA development. Despite an ever-growing interest in the interactions taking place at the human host–associated microbiome–pathogens interface in autoimmune disorders, only a limited number of studies investigated a possible association between *Mycoplasma* infection and RA (14–17).

Among mycoplasmas, *M. hominis* is a urogenital bacterium that can be associated with a number of conditions. Beyond infections, there is some evidence suggesting a potential role of *M. hominis* in inducing specific autoimmune disorders in susceptible subjects. First, *M. hominis,* similar to many *Mycoplasma* species (18), is equipped with a surface nuclease which induces both NETosis and NETs degradation *in vitro* (19). Furthermore, *M. hominis* energy metabolism relies mainly on the activity of an arginine deiminase pathway, which leads to the formation of arginine (20) and could therefore contribute to ACPA generation, manipulating immune response, disrupting host immune tolerance, and favoring the development of autoimmune diseases, including RA. A further clue in this direction comes from the *M. hominis*-induced IL-23 production by human dendritic cells (21).

This proposition is supported by studies in mice infected with *M. hominis* where the animals produced scleroderma-specific autoantibodies directed against centrosomes (22). Moreover, a higher prevalence of *M. hominis* was reported in the urine of women with systemic lupus erythematosus compared with the general population (23).

Given the relative paucity of data regarding the potential pathophysiological role of *M. hominis* in RA, we sought to investigate the presence and prevalence of antibodies against *M. hominis* lipid-associated membrane proteins (LAMPs) in RA patients and a control population of healthy subjects from two independent centers (Italy and Vietnam), and evaluate potential correlations between humoral responses and RA-specific clinical and serological variables.

We enrolled a consecutive series of unselected RA patients classified according to the ACR-EULAR (European League Against Rheumatism/American College of Rheumatology) 2010 international criteria (6), attending the outpatient clinics of the UO Reumatologia-Azienda Ospedaliero-Universitaria di Sassari (Italian cohort) and the Hospital of Hue University of Medicine and Pharmacy (Hue UMP) (Vietnamese cohort). Age- and sex-matched healthy controls were recruited from the staff of the Department of Biomedical Sciences, University of Sassari, and from Hue UMP, respectively. The study was approved by the local Ethics Committees (Italy: authorization PG-2019/8864, Vietnam: authorization H2020/022), and all participants signed an informed consent.

In the Italian series, the following data were also extracted from clinical records: C-reactive protein (CRP); erythrocyte sedimentation rate (ESR); disease activity index 28 joints with ESR (DAS28-ESR); positivity for the rheumatoid factor (RF); positivity for ACPA; use of steroids; and use of conventional disease-modifying anti-rheumatic drugs (csDMARDs), biologics, and targeted synthetic disease-modifying anti-rheumatic drugs (tsDMARDs). In the Vietnamese series, only the data on the use of immunosuppressants were available for extraction. (Table 1).

**TABLE 1:** Values are mean ± SD or median (IQR)^*a*^^*,^b^*^

	Italian cohort			Vietnamese cohort		
	RA (*n* = 66)	Controls (*n* = 66)	*P-*value	RA (*n* = 42)	Controls (*n* = 80)	*P-*value
Age, years	65.4 ± 10.2	65.7 ± 11.1	0.88	55.5 ± 12.1	39.4 ± 17.0	<0.001
Gender, F/M	43/23	44/22	0.85	37/5	47/33	0.001
CRP, mg/dL	0.49 (0.95)			-		
ESR, mm/h	21 (24)			-		
DAS28-ESR	3.3 (1.83)			-		
RF, %	66.1			-		
ACPA, %	57.1			-		
Steroid use, %	69.7			21.4		
Metotrexate use, %	53.8			50.0		
Leflunomide use, %	12.1			-		
Hydroxychloroquine use, %	18.4			-		
Tocilizumab use%	13.6			42.8		
TNFi use, %	21.2			-		
Abatacept use, %	12.1			-		
JAKi use, %	3.0			-		
No-treatment	9.0			14.2		
Anti-LAMPs, OD	0.087 (0.07)	0.052 (0.05)	<0.001	0.164 (0.07)	0.119 (0.02)	<0.001

^*a*^ ACPA, Anticitrullinated cyclic peptide antibodies; CRP, C-reactive protein; DAS28-ESR, Disease Activity Score-28 ESR; DMARDs, disease modifying antirheumatic drugs; ESR, Erythrocyte Sedimentation Rate; TNFi, Tumor Necrosis Factor-inhibitors; JAKi, Janus Kinases-inhibitors; Anti-LAMPs, anti-Lipid Associated Membrane Proteins ; OD, optical density.

^*b*^ “-”, stands for "not present" or not detected.

*M. hominis* reference strain PG21 was routinely cultivated in BE medium (25). LAMPs were purified by Triton X-114 phase separation (24) from cells in exponential phase growth indicated by medium color change, quantified by BCA method, and then used to coat 96-well enzyme-linked immunosorbent assay (ELISA) plates (150 ng LAMPs/well). Serum samples were diluted 1:100 and tested for LAMPs reactivity by incubating plates with goat polyclonal anti-human antibodies (α, γ, μ-chain specific). Immune complexes were detected by measuring the *p*-nitrophenyl phosphate (Sigma-Aldrich) color change at 405 nm.

A Mann–Whitney U test was used to analyze nonparametric data and compare differences in concentrations of anti-LAMPS antibodies between RA patients and HC. The ROC (receiver operating characteristic) curves were calculated, and the Youden’s *J* statistics was used to identify the optimal cut-off value. Differences in the prevalence of antibodies between the groups were then evaluated using the χ^2^ test. Statistical analyses were performed using Stata 18 and GraphPad Prism softwares. Statistically significant difference was set at *P* < 0.05.

Anti-*M*. *hominis* antibodies were detected in RA patients’ sera compared to an HC population, with significant differences in both optical densities (OD) and antibody detection between the two groups (*P* < 0.05).

**Fig 1:**
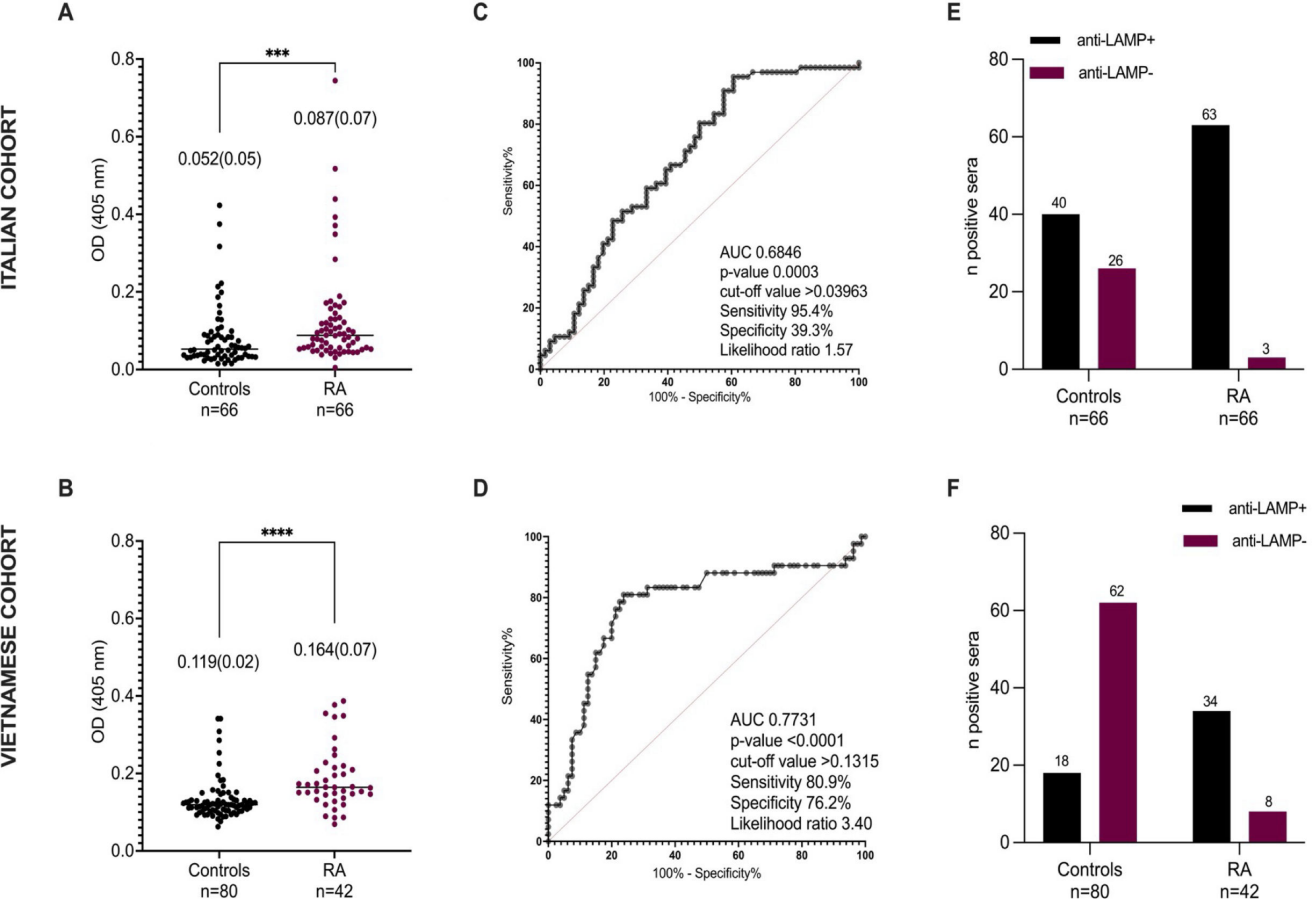
Anti-LAMPS antibodies in RA patients and controls. (**A** and **B**) OD values of anti-LAMPS antibodies were higher in RA patients when compared to HC in both the Italian and Vietnamese cohorts [0.087 (0.07) vs 0.052 (0.05) optical density (OD), respectively, in the Italian cohort, *P* < 0.001; 0.164 (0.07) vs 0.119 (0.02) OD, respectively, in the Vietnamese cohort, *P* < 0.001]. (**C and D**) ROC curves. (**E** and **F**) anti-M. *hominis* seropositivity/negativity proportions in RA and control populations *P < 0.0001.

**Fig 2:**
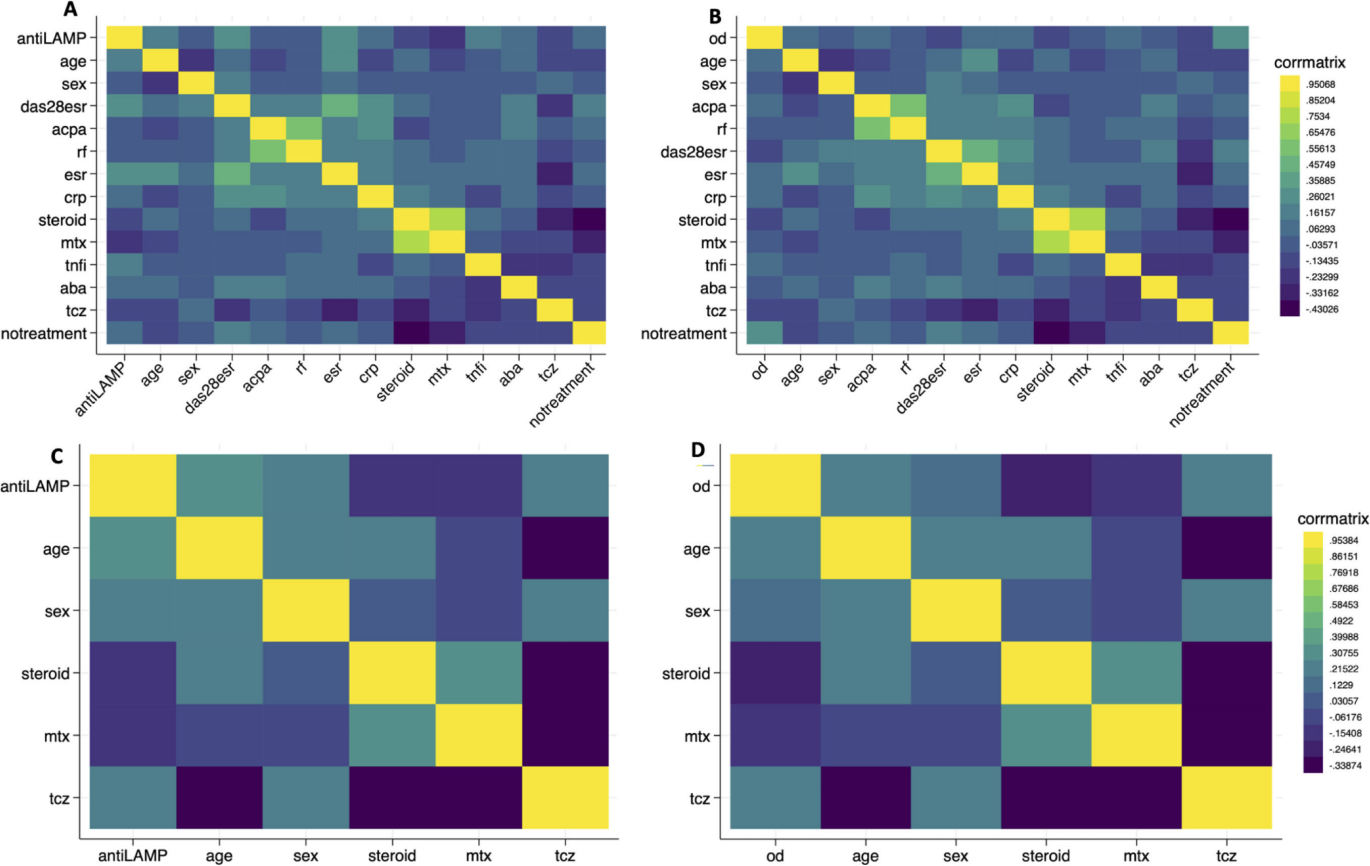
Correlation matrices. Italian (**A and B**) and Vietnamese (**C and D**) cohorts. j ACPA, Anticitrullinated cyclic peptide antibodies; CRP, C-reactive protein; DAS28-ESR, Disease Activity Score-28 ESR; ESR, Erythrocyte Sedimentation Rate; HCQ, hydroxychloroquine; RF, rheumatoid factor; TNFi, Tumor Necrosis Factor-inhibitors; JAKi, Janus Kinases-inhibitors; Anti-LAMPs, anti-lipid associated membrane proteins; OD, optical density.

Based on ROC-derived cut-off values, 95.4% of RA patients vs 60.6% of HC showed immunoreactivity against LAMPS of *M. hominis* in the Italian cohort. Similarly, in the Vietnamese cohort, 80.9% of RA sera were anti-LAMPS antibodies positive compared with only 22.5% of HC sera (see Fig. 1E and F). In bivariate correlation analysis, no significant correlations were identified between the OD_405 nm_ values or the positivity of anti-LAMPs antibodies and clinical and serological RA variables in both the Italian and Vietnamese cohorts (Fig. 2).

In line with our observations, several studies have reported the presence of a specific humoral response towards immunogenic peptides derived from bacteria belonging to the genus *Mycoplasma* in patients with different autoimmune diseases (5, 23).

Compelling, reliable, and replicated evidence in two independent RA cohorts differing for ethnic and clinical variables represents the main strength of our study, supporting a hypothetical role of *M. hominis* as a microbial trigger in RA pathogenesis. However, some limitations deserve clarification: whether RA condition and *M. hominis* serostatus are indeed linked by a cause–effect relationship, or is merely a cophenomenon, needs more mechanistic studies aimed at elucidating the potential contribution of *M. hominis* in RA onset and progression. Furthermore, it should be taken into account that the vast majority of sera were from RA patients who were being treated with immunosuppressive drugs. Therefore, we cannot completely discharge the assumption that concomitant immunosuppression may favor *M. hominis* infection in RA patients, inducing an increase in anti-LAMPs antibodies prevalence. However, this therapy could also account for a general poor humoral immune response leading to an underestimate of *M. hominis* seroprevalence in these subjects. Finally, in our study, the presence of specific anti-LAMPS antibodies was not significantly associated with ACPA positivity and disease activity, suggesting that the response against *M. hominis* could be related to unexplored factors independent of RA. Also of note, a significant linear relationship was found between age and OD values (the OD value increases by 0.007 per decade of age). However, when controlling for age, the origin of the samples also exerts an independent effect, with higher OD values observed in Vietnamese sera (data not shown). This discrepancy could be attributable to geographic, epidemiological, ethnic, or therapeutic factors, which unfortunately cannot be further explored in this study. Nevertheless, these results encourage further investigation of the possible role of *M. hominis* in RA pathogenesis via its concomitant NETs-inducing and NETs-disrupting activities.
